# The core tendencies underlying prosocial behavior: Testing a person–situation framework

**DOI:** 10.1111/jopy.12957

**Published:** 2024-07-02

**Authors:** Natalie Popov, Isabel Thielmann

**Affiliations:** ^1^ Department of Criminology Max Planck Institute for the Study of Crime, Security and Law Freiburg Germany

**Keywords:** bifactor modeling, economic games, personality, person–situation interaction, pro‐social behavior, situational affordances

## Abstract

**Objective and Background:**

According to a recently proposed theoretical framework, different personality traits should explain pro‐social behavior in different situations. We empirically tested the key proposition of this framework that each of four “core tendencies” (i.e., the shared variance of related traits) specifically predicts pro‐social behavior in the presence of a different situational affordance.

**Methods:**

We used a large‐scale dataset (*N* = 2479) including measures of various personality traits and six incentivized economic games assessing pro‐social behavior in different social situations. Using bifactor modeling, we extracted four latent core tendencies and tested their predictive validity for pro‐social behavior.

**Results:**

We found mixed support for the theoretically derived, preregistered hypotheses. The core tendency of beliefs about others' pro‐sociality predicted pro‐social behavior in both games involving dependence under uncertainty, as expected. Unconditional concern for others' welfare predicted pro‐social behavior in only one of two games providing a possibility for exploitation. For conditional concern for others' welfare and self‐regulation, in turn, evidence relating them to pro‐social behavior in the presence of a possibility for reciprocity and temporal conflict was relatively weak.

**Conclusion:**

Different features of social situations may activate different personality traits to influence pro‐social behavior, but more research is needed to fully understand these person–situation interactions.

## 1 INTRODUCTION

Prosocial behaviors have positive consequences for individuals and society at large, promoting social harmony and trust, and reducing conflict and aggression (Balliet et al., 2022; Dovidio et al., 2017; Haller et al., 2021; Nowak, 2006). Broadly defined, pro‐social behavior involves “any action that benefits another person” (Schroeder & Graziano, 2015, p. 255). Examples include donating to those in need, refraining from stockpiling when resources are scarce, or taking public transportation to protect the environment. Given this ubiquity, studying the underlying factors of prosocial behavior receives perennial interest in the social sciences and beyond (Besley & Ghatak, 2018; Simpson & Willer, 2015; Spadaro et al., 2022; Thielmann & Pfattheicher, 2022).

Decades of research on human pro‐sociality have shown that individuals differ in their tendency to engage in prosocial behaviors: Some individuals often act prosocially; others primarily act in their own interest. These individual differences can well be accounted for by personality traits, that is, individuals' “relatively enduring patterns of thoughts, feelings, and behaviors that reflect the tendency to respond in certain ways under certain circumstances” (Roberts, 2009, p. 140). Indeed, various personality traits yield consistent relations with pro‐social behavior (Thielmann et al., 2020).

Theoretically, whether a certain personality trait will be related to pro‐social behavior in a given situation should depend on the features of that situation. This basic assumption is at the heart of trait–situation interactionism (Funder, 2009; Rauthmann et al., 2015; Zhao & Smillie, 2015) and has recently also been specifically applied in a theoretical framework of individual differences in pro‐social behavior (Thielmann et al., 2020). Although some conjectures of this framework have already been tested meta‐analytically based on correlations of 51 personality traits with pro‐social behavior in different situations (Thielmann et al., 2020), several open questions remain, thus calling for additional empirical tests. With the current work, we aim to provide such a test to further improve our understanding about which personality traits relate to pro‐social behavior in which situations. Using a large‐scale study involving 21 personality traits and measuring consequential behavior in six game‐based social situations, we test the theoretical proposition that four classes of personality traits can be distinguished, each of which uniquely predicts pro‐social behavior in the presence of different situational features.

### 1.1 A theoretical framework of individual differences in pro‐social behavior

Integrating the diverse evidence linking personality to pro‐social behavior, Thielmann et al. (2020) recently proposed a theoretical framework specifying which classes of personality traits should be related to pro‐social behavior under which circumstances. The basic premise of the framework is that different situational features—so‐called *affordances—*allow different personality traits to become expressed in pro‐social behavior. In general, situational affordances denote “properties of the situation that provide a context for the expression of motives, goals, values, and preferences” (Reis, 2008, p. 316). To illustrate, consider a self‐serve coffee shop where customers are trusted to pay for the items they take without monitoring them. In this situation, customers have the choice between getting something without paying and paying the appropriate amount for the items they chose. Thus, the situation involves a possibility for exploitation: A person may simply act in their own interest and exploit the trust‐based system. However, this comes at the potential cost for other costumers because the shop may eventually close if everyone acts in the same (selfish) way.

As proposed in the affordance‐based framework of individual differences in pro‐social behavior, four key situational affordances primarily characterize interdependent situations. Interdependent situations describe any social situations in which the outcomes of involved parties depend on each other's choices. The four affordances are (i) a possibility for exploitation, (ii) a possibility for reciprocity, (iii) temporal conflict between short‐term and long‐term interests, and (iv) dependence on others under uncertainty.^1^ Each of these affordances should, in turn, activate certain personality traits and thereby allow their expression in pro‐social versus selfish behavior. Table 1 provides an overview of the four affordances proposed, together with the trait classes that should be activated in respective situations.

**TABLE 1:** Key situational affordances for prosocial behavior provided in interdependent situations as proposed by Thielmann et al. (2020).

Affordance	Situational features	Allows the expression of …	
		Trait class	Narrow traits
(i) Possibility for exploitation	Individuals can increase their own outcome at others' expense, particularly if there is no punishment for selfish behavior	Unconditional concern for others' welfare	For example, altruism, empathy, exploitativeness (−)
(ii) Possibility for reciprocity	Individuals can react to others' (pro‐social vs. selfish) behavior by either returning a favor (positive reciprocity) or by refraining from retaliation (negative reciprocity)	Conditional concern for others' welfare	For example, forgivingness, negative reciprocity (−), vengefulness (−)
(iii) Temporal conflict	Individuals are faced with a conflict between their own short‐term self‐interests and long‐term (collective) interests	Self‐regulation	For example, consideration of future consequences, impulsivity (−), self‐control
(iv) Dependence under uncertainty	Individuals' outcome depends on others' unknown behavior	Beliefs about others' pro‐sociality	For example, beliefs in reciprocity, cynicism (−), trust propensity

*Note*: (−) indicates a negative relation to pro‐social behavior.

First, a *possibility for exploitation* is present whenever an individual can increase their own outcome at others' expense, particularly so if individuals do not have to fear sanctions for selfishness. The coffee shop example above illustrates this nicely. Another example for a possibility for exploitation is a supervisor who can freely take credit for a collaborative work with a subordinate without having to fear negative repercussions. Theoretically, situations characterized by a possibility for exploitation allow for the expression of traits related to *unconditional concern for others' welfare*, that is, the tendency to benevolently consider others' outcomes in one's own decisions. Traits capturing unconditional concern are, for example, altruism, empathy, and (low) exploitativeness (for definitions, see Table 2).

**TABLE 2:** Personality traits included for each trait class.

Trait class	Construct	Definition	Questionnaire	*N* items	Example item
Unconditional concern for others' welfare	Altruism	“Sharing, helping, taking care of, and feeling emphatic with others and their needs or requests” (Batson et al., 1986, p. 212)	Pro‐socialness Scale (Caprara et al., 2005)	16	I try to help others
	Compassion	“Attitude toward other(s), either close others or strangers of all of humanity; containing feelings, cognitions, and behaviors that are focused on caring, concern, tenderness, and an orientation toward supporting, helping, and understanding the other(s)” (Sprecher & Fehr, 2005, p. 630)	Santa Clara Brief Compassion Scale (Hwang et al., 2008)	5	I would rather engage in actions that help others, even though they are strangers, than engage in actions that would help me
	Empathy	“Comprehension of other people's experience (cognitive empathy) as well as the ability to vicariously experience the emotional experience of others” (affective empathy) (Reniers et al., 2011, p. 85)	Interpersonal Reactivity Index (Davis, 1980)	14	I often have tender, concerned feelings for people less fortunate than me
	Exploitation	“The state, condition, quality, or degree of unfairly or cynically using another person or group for profit or advantage” (Brunell et al., 2013, p. 2)	Interpersonal Exploitativeness Scale (Brunell et al., 2013)	6	It does not bother me to benefit at someone else's expense
	Selfishness—egocentric	“Selfishness with a single‐minded attentional focus on the self” (Raine & Uh, 2019, p. 504)	Selfishness Questionnaire (Raine & Uh, 2019)	8	I care for myself much more than for others
	Selfishness—adaptive	“A ‘softer’ form of selfish behavior with an eye to others” (Raine & Uh, 2019, p. 503)	Selfishness Questionnaire (Raine & Uh, 2019)	8	I mostly help those around me who will help me later
	Social welfare concerns	“The extent to which people are concerned with the welfare of our society in general” (Haesevoets et al., 2018, p. 423)	The Self‐ and Other‐Interest Inventory (Gerbasi & Prentice, 2013)	3	I am concerned with the overall best interest for everyone
Conditional concern for others' welfare	Aggressiveness (facet: hostility)	“Hostility, which consists of feelings of ill will and injustice, represents the cognitive component of [aggressive] behavior” (Buss & Perry, 1992, p. 457)	Buss–Perry Aggression Questionnaire (Buss & Perry, 1992)	8	I am sometimes eaten up with jealousy
	Forgiveness	“Disposition to forgive interpersonal transgressions over time and across situations” (Berry et al., 2005, p. 183)	Trait Forgivingness Scale (Berry et al., 2005)	10	I can forgive a friend for almost anything
	Neg. reciprocity norm endorsement	“Beliefs favoring the reciprocation of unfavorable treatment” (Eisenberger et al., 2004, p. 787)	Negative Reciprocity Norm Endorsement Scale (Eisenberger et al., 2004)	14	If someone dislikes you, you should dislike them
	Positive reciprocity	“Reactions to positively valued behaviors with the emphasis on rewarding someone else's behavior” (Perugini et al., 2003, p. 274)	Personal Norm of Reciprocity Scale (Perugini et al., 2003)	9	If someone does a favor for me, I am ready to return it
	Vengefulness	“Disposition that orients people toward revenge after they have suffered an interpersonal offense” (McCullough et al., 2001, p. 602)	Vengeance Scale, short (Coelho et al., 2018; Furr & Funder, 2004)	10	It is important for me to get back at people who have hurt me
Self‐regulation	Consideration of future consequences	“Stable individual difference in the extent to which people consider distant versus immediate consequences of potential behaviors” (Strathman et al., 1994, p. 742)	Consideration of Future Consequences Scale‐14 (Joireman et al., 2012)	12	My behavior is generally influenced by future consequences
	Impulsivity	“Tendency to deliberate less than most people of equal ability before taking action” (Dickman, 1990, p. 95)	Barrat Impulsiveness Scale BIS‐15 (short form: Spinella, 2007)	15	I do things without thinking
	Self‐control	“Ability to override or change one's inner responses, as well as to interrupt undesired behavioral tendencies (such as impulses) and refrain from acting on them (Tangney et al., 2004, p. 274)	Brief Self‐Control Scale (Sproesser et al., 2011)	13	I am good at resisting temptation
	Social responsibility	[Willingness to follow the] “norm prescribing that the individual should help those who are dependent upon [them]” (Berkowitz & Daniels, 1964, p. 275)	Social Responsibility Scale (Berkowitz & Daniels, 1964)	8	I am the kind of person that people can count on
Beliefs about others' pro‐sociality	Cynicism	“A general attitude of contempt or skepticism about human beings and their values…[belief] belief that everyone has a price; that ideals are easily shown up to be empty when they conflict with self‐interest” (Vice, 2011, p. 172)	Cynicism scale (Chowdhury & Fernando, 2014)	5	People pretend to care more about one another than they really do
	Dangerous world view	“Belief that the social world is a dangerous and threatening place in which good, decent people's values and way of life are threatened by bad people versus belief that the social world is a safe, secure and stable place in which almost all people are fundamentally good” (Perry et al., 2013, p. 125)	Two World View Scale (Sibley & Duckitt, 2009)	6	There are many dangerous people in our society who will attack someone out of pure meanness, for no reason at all
	General trust	“General willingness to trust others” (Mayer et al., 1995, p. 714) [based on the] “expectation of partner's goodwill and benign intent” (Yamagishi & Yamagishi, 1994, p. 131)	General Trust Scale (Yamagishi & Yamagishi, 1994)	6	Most people are basically honest
	Reciprocity beliefs	“View that both forms of [positive and negative] reciprocity are generally effective and widely used” (Perugini et al., 2003, p. 274)	Personal Norm of Reciprocity Scale (Perugini et al., 2003)	9	If I work hard, I expect it will be repaid
	Trust propensity	“General willingness to trust others regardless of social and relationship‐specific information” (Frazier et al., 2013, p. 77)	Trust Propensity Scale (Frazier et al., 2013)	4	I usually trust people until they give me a reason not to trust them

Second, a *possibility for reciprocity* is present whenever individuals react to others' pro‐social versus selfish behavior. For instance, if the subordinate in the previous example later has the opportunity to take revenge on their supervisor, they encounter a possibility for (negative) reciprocity. If the supervisor, by contrast, did give full credit to the subordinate, the subordinate encounters a possibility for positive reciprocity, that is, to return the favor. Situations involving a possibility for reciprocity should allow the expression of traits related to *conditional concern for others' welfare*, that is, the tendency to consider others' outcomes in one's reactions to their previous behavior. Traits capturing the high pole of conditional concern for others are, for example, forgivingness, (low) negative reciprocity, and (low) vengefulness.

Third, a *temporal conflict* between short‐term and long‐term interests is present when the pursuit of immediate benefits conflicts with the pursuit of long‐term benefits. In the previous example, the supervisor might be torn between maximizing short‐term benefits by pressuring their subordinates without regard for their well‐being and adopting sustainable work practices that may yield long‐term benefits for both the company and its employees—and, thereby, also the supervisor. Situations characterized by temporal conflict allow for the expression of traits related to *self‐regulation*, that is, personality traits that capture individuals' the tendency to refrain from giving in on one's impulses in favor of considering the long‐term consequences of one's actions, as, for example, captured by consideration of future consequences, self‐control, and (low) impulsivity.

Fourth, *dependence on others under uncertainty* is present whenever an individual's own outcome is conditional on others' unknown behavior. This is the case whenever an individual does not have full power over their final outcome and they do not know what their interaction partners will do. In the above example, the subordinate would face dependence under uncertainty if they trusted the supervisor to correctly communicate both individuals' involvement in the project. Situations involving dependence under uncertainty allow for the expression of *beliefs about others' pro‐sociality*, that is, individuals tendency to think that others are trustworthy versus untrustworthy. Such beliefs are, for example, captured by traits such as (low) cynicism, beliefs in reciprocity, and trust propensity.

In summary, according to the framework tested here, four key affordances can be distinguished in interdependent situations, each of which activates different personality traits to be expressed. Providing preliminary support for this framework, a meta‐analysis based on 770 studies showed that the overall pattern of correlations between 51 personality traits and pro‐social behavior largely corresponded to the hypothesized associations (Thielmann et al., 2020). Specifically, the meta‐analysis included studies measuring pro‐social behavior in six different economic games. Games are an established tool to assess pro‐social behavior in controlled, experimental settings (Thielmann et al., 2021; van Dijk & de Dreu, 2021). A key advantage of economic games is that they can be implemented with real (e.g., monetary) incentives to render decisions consequential, that is, participants and their interaction partners are incentivized according to each other's behavior. Moreover, knowing the type and structure of a game allows one to derive which situational affordances are present and, therefore, which (classes of) personality traits should guide behavior. For example, in the Dictator Game, a dictator can choose how much, if any, of a given endowment to share with a recipient (Forsythe et al., 1994). The more the dictator gives, the more pro‐social their decision is considered to be. Given that the recipient cannot react to the dictator's decision, the Dictator Game is a prime example of a situation involving a possibility for exploitation for dictators. In turn, in line with the framework's predictions, several personality traits capturing individual differences in unconditional concern for others' welfare showed significant, small to medium‐sized relations with Dictator Game giving. Conversely, these traits showed (descriptively) weaker correlations with behavior in other games in which the possibility for exploitation is less prominent or entirely absent. Moreover, traits from other trait classes (e.g., conditional concern for others' welfare) showed largely weaker relations with Dictator Game giving than those capturing unconditional concern for others' welfare.

### 1.2 Limitations of prior research

Although the meta‐analysis by Thielmann et al. (2020) provides initial support for the proposed affordance‐based framework, the conclusiveness of findings is limited. First, certain trait classes and affordances were underrepresented in the meta‐analysis. For example, out of all 51 traits included, only four were identified as capturing individual differences in beliefs about others' pro‐sociality. Inferences about this trait class thus strongly depend on the intricacies of the specific traits examined and generalizing these findings to the trait class as a whole may not be warranted. Similarly, the affordance of temporal conflict was underrepresented and mostly secondary in the economic games included in the meta‐analysis. This may explain why particularly those traits capturing individual differences in self‐regulation showed (very) weak relations with pro‐social behavior at best.

Moreover, the conception of different trait classes as proposed by the affordance‐based framework is justified solely on theoretical grounds. The extent to which personality traits assigned to the same trait class actually share a conceptual *common core* (referred to as “psychological process” by Thielmann et al., 2020) that may drive individual differences in corresponding behavior has not been tested yet. The idea of a common core suggests that it cannot be adequately operationalized by a single trait, let alone a single scale. Instead, the common core will capture aspects from multiple traits from the same trait class to broadly reflect the respective theoretical construct. Analogous to the logic underlying the “dark factor” of personality (Moshagen et al., 2020), which represents the common core of all dark traits, we conceptualize the four core tendencies as fluid constructs. Thus, each core tendency is supposed to represent the primary source of variance in individual differences in the traits from the respective trait class. Consequently, any instrument measuring a specific trait from the trait class related to a core tendency should also reflect the core tendency to a certain degree. In the present study, we therefore included multiple trait measures per trait class and extracted their common core using latent variable modeling to predict pro‐social behavior.

### 1.3 The present research

In a preregistered study, we provide a direct empirical test of the affordance‐based framework of individual differences in pro‐social behavior. To this end, we relied on data from the Pro‐social Personality Project (PPP; Thielmann et al., 2022; https://osf.io/m2abp/), a multi‐wave study measuring various personality traits alongside pro‐social behavior in economic games in a large (*N* = 2258) and demographically diverse sample. As such, the PPP provided a unique testbed for the present purpose, overcoming key limitations of prior research.

First, the PPP includes various personality traits that were specifically selected so as to broadly capture the four trait classes proposed in the affordance‐based framework. Thus, unlike studying the influence of single traits on pro‐social behavior separately, the data allowed us to extract the commonalities of traits from the same trait class and to correlate the resulting common core (i.e., “core tendency”) with pro‐social behavior in different social situations. To this end, we applied structural equation modeling (SEM), specifically bifactor modeling. A key advantage of this approach is that we do not capitalize on the intricacies of specific traits or scales, respectively, but rather capture each trait class more broadly.

Second, the PPP includes measures of pro‐social behavior in six situations modeled in different, commonly used economic games: Trust Game (as trustor and trustee), Public Goods Game, Spite Game (as proposer and responder), and Volunteer's Dilemma. These games allow unveiling specific aspects of pro‐social behavior (Thielmann et al., 2015; van Dijk & de Dreu, 2021). In fact, the games included in the PPP were specifically selected so as to primarily provide one of the four key situational affordances proposed to be present in interdependent situations. This allowed us to test whether each core tendency will indeed account for behavior in the presence of the respective affordance, as hypothesized.

### 1.4 Hypotheses

Based on the above reasoning, we expected that individual differences in pro‐social behavior are the result of certain (classes of) personality traits being expressed in response to certain situational affordances. Specifically, as per the affordance‐based framework (Thielmann et al., 2020), the hypotheses are as follows.

1. The core tendency reflecting unconditional concern for others' welfare will be more strongly associated with pro‐social behavior in situations providing a possibility for exploitation, specifically in the Trust Game as trustor (H1a) and the Public Goods Game (H1b), compared to pro‐social behavior in situations involving other affordances.
2. The core tendency reflecting conditional concern for others' welfare will be more strongly associated with pro‐social behavior in situations providing a possibility for reciprocity, specifically in the Trust Game as trustee (H2a) and the Spite Game as responder (H2b), compared to situations involving other affordances.
3. The core tendency reflecting self‐regulation will be more strongly associated with pro‐social behavior in situations characterized by a temporal conflict between short‐term and long‐term interests, specifically in the Spite Game as proposer (H3a) and the Volunteer's Dilemma (H3b), compared to situations involving other affordances.
4. The core tendency reflecting beliefs about others' pro‐sociality will be more strongly associated with pro‐social behavior in situations involving dependence under uncertainty, specifically in the Trust Game as trustor (H4a) and the Public Goods Game (H4b), compared to situations involving other affordances.

## 2 METHODS

The hypotheses and analysis plan were preregistered prior to conducting the main analyses (see https://osf.io/cymxz) https://tinyurl.com/28u2tnuw). Any deviations from the preregistration are summarized on the Open Science Framework (OSF), where we also provide the data and analyses scripts (https://osf.io/cymxz). (https://tinyurl.com/38p548wv).

### 2.1 Participants and procedure

Participants for the PPP were recruited online via a panel provider in Germany. A detailed documentation of the PPP including information on all measures, sample sizes, and compositions per wave, a priori specified exclusion criteria, and previous publications using (subsets of) the data are available on the OSF (https://osf.io/m2abp) (https://tinyurl.com/2x2u3rnf). A total of *N* = 4585 participants (51.4% female, aged 18–78 years, *M* = 40.2, *SD* = 13.0) comprised the final sample at wave 1 and were re‐invited for participation in the subsequent waves. For the present study, we used a subset of data collected at wave 3 (conducted 61 days after wave 1 on average), wave 4 (conducted 84 days after wave 1 on average), wave 5 (conducted 110 days after wave 1 on average), and wave 6 (conducted 132 days after wave 1 on average). Waves 3–5 each included multiple personality traits from the four trait classes as per the affordance‐based framework, that is, traits capturing individual differences in unconditional concern for others' welfare, conditional concern for others' welfare, self‐regulation, and beliefs about others' pro‐sociality (see Table 2). Wave 6 included multiple economic games to measure pro‐social behavior while participants were randomly assigned to complete only one of these games. To render game decisions truly consequential, participants were randomly matched with other participants according to the rules of a game after completion of data collection. Based on the matched participants' decisions, participants received behavior‐contingent, monetary incentives that were paid out anonymously by the panel provider.

### 2.2 Measures

#### 2.2.1 Personality traits

Table 2 provides an overview of the 21 personality traits considered in the current study, along with the scale used to measure each trait. Participants' responses were recorded on five‐point Likert‐type scales ranging from 1 = “strongly disagree” to 5 = “strongly agree.”

Although the personality traits measured at waves 3–5 of the PPP were specifically selected to capture the four trait classes, we reevaluated the allocation of each trait to a trait class to select only those traits showing a unique conceptual link to the respective trait class.^2^ To this end, both authors independently inspected the definitions and operationalizations (i.e., items) of each trait to determine whether the trait in question indeed unequivocally tapped into the trait class it was originally assigned to. Subsequently, we determined the strength of overlap between the identified traits per trait class. For the bifactor models, it is important that indicators of the same latent trait share meaningful variance to allow extracting a strong common core, if present, and thereby produce reliable parameter estimates. Thus, we first examined the (zero‐order) correlations between the traits selected per trait class and excluded those traits showing only small average correlations with the remaining traits of that class. Moreover, we ran confirmatory factor analyses (CFA) on each trait scale to evaluate whether the items of the same scale shared meaningful variance and, thus, whether the latent factors of the unidimensional models for each trait scale were well represented by their indicators. We excluded a trait scale if the majority of respective item loadings fell below *λ* = 0.4. Overall, this procedure ensured that the traits selected to represent a specific core tendency were effectively characterized by a shared common variance captured by the general factor and specific variance unique to each trait.

#### 2.2.2 Economic games

##### Trust Game

The Trust Game (Berg et al., 1995) involves two players, the trustor and the trustee. Both players received an initial endowment of 3€. Trustors decided how much of their 3€ (in 0.50€ increments), if any, to send to the trustee. Transfers were tripled, thus increasing social welfare. Trustees decided how much of the tripled transfer, if any, to return to the trustor (again in 0.50€ increments). Participants completing the Trust Game were randomly assigned to the role of either the trustor or the trustee. Given that trustors and trustees completed the study simultaneously, trustee responses were elicited using the strategy method (Selten, 1967). That is, trustees reported their return for each possible transfer by the trustor (between 0.50€ and 3€) and we computed the average return across the six decisions as a measure of pro‐social behavior. In terms of affordances, trustors face a possibility for exploitation because they can decide to simply keep their endowment and not share anything with the trustee, in which case the trustee does not have any say about the final distribution of outcomes, as well as dependence under uncertainty because trustors do not know what the trustee will do in case they transfer anything. Trustees, in turn, encounter a possibility for reciprocity because they can react to the trustor's (anticipated) transfer.^3^

##### Public Goods Game

In the Public Goods Game (Samuelson, 1954), participants were assigned to groups of four. Each group member received an endowment of 4€ and decided independently of each other how much of it (in 0.50€ increments), if any, to contribute to a group account. Contributions to the group account were doubled—so as to increase social welfare—and equally distributed among all group members, independent of each member's individual contribution. Thus, each group member can maximize their individual outcome by not contributing to the group account while profiting from other group members' contributions. As such, the Public Goods Game provides a possibility for exploitation. At the same time, it also involves dependence on others under uncertainty because group members do not know what others do.

##### Spite Game

The Spite Game involves two players, the proposer and the responder (Güth & Huck, 1997), to which participants were randomly assigned. Proposers received 5€ and were asked how much of this endowment (in 0.50€ increments), if any, to share with the responder, which defined their offer. Responders decided whether to accept or reject the proposer's offer. If the responder in the Spite Game accepts the offer, both players receive the amount as distributed by the proposer. However, if the responder rejects, the proposer goes away empty handed while the responder still receives their share as allocated by the proposer. Given that proposers and responders took part in the study simultaneously, responders indicated the smallest offer between 0€ and 5€ they would accept (i.e., the minimum acceptable offer), meaning that they would reject any offer below the specified amount. Thus, the lower the minimum acceptable offer, the more pro‐social one's choice. Overall, responders can react to the proposer's anticipated offer and reduce the proposer's payoff at no cost to themselves. Thus, responders encounter a possibility for (negative) reciprocity. Proposers, by contrast, face a temporal conflict between short‐term self‐interest and long‐term benefits for both players: If proposers solely consider the first part of the game (i.e., their own choice) while neglecting the responder's possibility to react, they may keep a large share or even the entire amount for themselves because it arguably increases their immediate outcome. However, if proposers consider responders' potential reactions, and thus the potentially negative long‐term consequences of being selfish in the first place, they may share a larger amount with the responder than they would prefer in order to increase their ultimate outcome.

##### Volunteer's Dilemma

In the Volunteer's Dilemma (Diekmann, 1985), participants were randomly assigned to groups of four. Each group member received 2€ and independently decided whether or not to invest this amount (i.e., to volunteer) for the group to secure 4€ for all other group members while ending up with 2€ themselves. Importantly, if no one volunteered, all group members ended up with 0€. Thus, group members face a temporal conflict because they have to decide between immediate personal costs and uncertain future benefits for the group. In other words, they may keep their endowment to potentially maximize their own outcome or invest in the group, thus suppressing their immediate (selfish) urge but benefitting everyone, including themselves, in the long run.

### 2.3 Statistical modeling and analysis

To model the core tendencies—that is, what all traits from the same trait class have in common—we resorted to bifactor modeling. In a bifactor model, the variances of manifest indicators (i.e., item responses) are decomposed into a general factor and multiple specific factors, one for each trait scale the items originate from. The general factor represents the common core, capturing what all items from the scales in a model have in common; the specific factors are orthogonal to (i.e., uncorrelated with) the general factor and capture the remaining shared variances of items from the same scale. We specified four baseline bifactor models, one for each trait class, with the general factor representing another core tendency in each model (i.e., unconditional concern for others' welfare, conditional concern for others' welfare, self‐regulation, and beliefs about others' pro‐sociality).

To evaluate model fit, we resorted to the robust root mean squared error of approximation (RMSEA) and the standardized root mean square residual (SRMR). In line with common conventions, RMSEA around 0.06 and SRMR around 0.08 indicate good fit to the data (Schermelleh‐Engel et al., 2003). For the sake of completeness, we further report the robust comparative fit index (CFI; e.g., Bentler, 1990). However, as this index relies more on the loading magnitude than on model misfit (Moshagen & Auerswald, 2018), we focus on RMSEA and SRMR. Further, to measure the degree with which each scale was subsumed by the common core (i.e., the general factor strength), we computed the explained common variance (ECV; Ten Berge & Sočan, 2004). The ECV quantifies the ratio of the common variance explained by the general factor to the entire variance explained by the general factor *and* the specific factors. Accordingly, the closer the ECV approaches 1, the stronger is the general factor. Model fit and ECV were evaluated for each of the four baseline bifactor models. As the baseline models do not include an outcome variable (i.e., game behavior), we used the complete non‐stratified sample available for the estimation, meaning that we included all cases with complete data on all measures from the same trait class. Importantly, given that not all participants completed all relevant waves of data collection (waves 3–5), sample sizes varied between the four baseline bifactor models (2501 ≤ *N* ≤ 2904).

To test our hypotheses, we used each general factor (i.e., core tendency) as a predictor of the two a priori assigned behavioral outcomes involving the respective affordance. For example, we used the general factor representing unconditional concern for others' welfare to predict behavior in those games involving a possibility for exploitation, that is, trustors' transfer in the Trust Game and contributions in the Public Goods Game. We ran separate latent regressions for each relevant outcome variable (i.e., two regressions per affordance or core tendency, respectively), each time including all participants who provided complete responses in the economic game at hand and on all relevant trait scales reflecting the respective trait class. The resulting proportion of explained variance (*R* ^2^) served as the effect size of interest. Following the widely accepted guidelines by Cohen (1992), we consider an *R* ^*2*^ greater than or equal to 0.025, which is equivalent to *r* = 0.15, as a meaningful association between the outcome variable and the general factor in the bifactor model.

All analyses were conducted in R (version 4.3.1; R Core Team, 2021) using the packages *lavaan* (version 0.6.16; Rosseel, 2012) and *BifactorIndicesCalculator* (version 0.2.2; Dueber, 2017). We applied maximum‐likelihood estimation with robust standard errors and scaled test statistics to account for potentially non‐normally distributed (Satorra & Bentler, 2001). For the purpose of model identification, we used a reference indicator for each specific factor whose factor loading was set to one. To examine differences in correlations as per our hypotheses, we compared the latent bivariate correlations using one‐sided *z*‐tests for comparing independent correlation coefficients (Meng et al., 1992) as implemented in the *cocor* package (version 1.1‐4; Diedenhofen & Musch, 2015). Note that this latter part of the analyses was not preregistered, even though our hypotheses implied significant differences in the strengths of correlations because we predicted each core tendency to *more strongly* correlate with behavior in some games compared to others.

## 3 RESULTS

### 3.1 Descriptive statistics

Descriptive statistics for all behavioral outcome measures are presented in Table 3. On average, participants transferred about half of their endowment in the Trust Game as trustor, in the Public Goods Game, and in the Spite Game as proposer. Similarly, trustees in the Trust Game returned half of the tripled transfer on average, whereas responders in the Spite Game requested only around two‐fifth of the proposer's endowment as offer to accept it. Finally, almost 80% of participants decided to volunteer in the Volunteer's Dilemma. Descriptive statistics and intercorrelations of the 21 personality trait measures are provided in the supplemental materials provided on the OSF (Tables S1–S5).

**TABLE 3:** Mean allocations and sample size for each economic game.

Game	*n*	Range	*M* (*SD*)	Primary situational affordance(s)
Trust Game (trustor)	452	0–3	1.66 (0.75)	Possibility for exploitation; dependence under uncertainty
Trust Game (trustee)	444	0–1	0.48 (0.18)	Possibility for (positive) reciprocity^a^
Public Goods Game	442	0–4	2.09 (0.95)	Dependence under uncertainty; possibility for exploitation
Spite Game (proposer)	391	0–5	2.55 (0.55)	Temporal conflict
Spite Game (responder)	410	0–5	1.91 (0.91)	Possibility for (negative) reciprocity
Volunteer's Dilemma	440	0;1	0.79 (0.41)	Temporal conflict

*Note*: *n* = number of participants completing the respective (role in) the economic game.

^a^ The trustee also faces a possibility for exploitation. We therefore exploratorily test the relation of trustee behavior with unconditional concern for others' welfare.

### 3.2 Baseline bifactor models of the core tendencies

As summarized in Table 4, all baseline bifactor models modeling the four core tendencies yielded acceptable to good fit to the data (0.052 ≤ RMSEA ≤ 0.071; 0.053 ≤ SRMR ≤ 0.909). The best‐fitting model occurred for conditional concern for others' welfare, followed by the models for unconditional concern for others' welfare and beliefs about others' pro‐sociality. The model for self‐regulation exhibited the lowest fit, which was, however, still in an acceptable range. In terms of the frequently used reliability indicator for bifactor models (i.e., coefficient omega *ω*; McDonald, 2013; Rodriguez et al., 2016), all core tendencies were highly reliable, thus indicating well‐defined latent constructs (i.e., 0.72 ≤ *ω* ≤ 0.90). Further details and additional model indices (e.g., factor determinacy; Grice, 2001) are available in the supplementary material (Table S6).

**TABLE 4:** Summary of model fit, proportion of explained variance by the general factor and factor loadings on the general factor for the four baseline bifactor models.

Core tendency	*n*	*df*	Model fit			ECV (%)	Loadings			
			RMSEA [90% CI]	SRMR	CFI		Mean	*SD*	Range	Item with strongest standardized loading
Unconditional concern for others' welfare	2865	1272 (159)	0.057 [0.056, 0.058]	0.063	0.842	64.6	0.52	0.13	0.02–0.79	“I am empathetic with those who are in need.” (*λ* = 0.79)
Conditional concern for others' welfare	2904	1173 (153)	0.052 [0.051, 0.053]	0.054	0.880	57.9	0.45	0.25	0.02–0.88	“If someone treats you badly, you should treat that person badly in return.” (*λ* = −0.88)
Self‐regulation	2751	1032 (144)	0.071 [0.070, 0.072]	0.072	0.711	48.5	0.38	0.13	0.13–0.67	“I am able to work effectively toward long‐term goals.” and “I concentrate easily.” (both *λ* = 0.67)
Beliefs about others' pro‐sociality	2501	380 (90)	0.056 [0.054, 0.057]	0.053	0.909	52.3	0.41	0.26	0.01–0.87	“I am trustful.” (*λ* = 0.87)

Abbreviations: CFI, (robust) comparative fit index; *df*, degrees of freedom (i.e., number of parameters); ECV, explained common variance; *n*, sample size; RMSEA, (robust) root mean square error of approximation; SRMR, standardized root mean square residual.

Moreover, there was considerable overlap among the traits used to model a core tendency. In all baseline bifactor models, the general factor explained at least around half of the variance in item responses (i.e., 48.5% ≤ *ECV* ≤ 64.6%). The highest proportion of variance explained by the general factor emerged for the model for unconditional concern for others' welfare, followed by the models of conditional concern for others' welfare, beliefs about others' pro‐sociality, and self‐regulation (Table 4).

Next, we inspected the relations among the general factor, specific factors, and unique variances in each model to see how common variances were distributed. A more detailed summary for the decomposition of the explained common variance for each specific factor is listed in the supplemental material (Table S7). For unconditional concern for others' welfare, the general factor captured the vast majority of item variances for empathy (*ECV*_*G*_ = 81.5%), compassion (*ECV*_*G*_ = 79.2%), and altruism (*ECV*_*G*_ = 78.5%). The general factor also accounted for the majority of common variance in items of the egocentric selfishness facet (*ECV*_*G*_ = 67.0%), whereas it accounted for less of the common variance in items of the adaptive selfishness facet (*ECV*_*G*_ = 35.0%). Explained item variances by the general factor were lowest for exploitativeness (*ECV*_*G*_ = 38.4%) and social welfare concerns (*ECV*_*G*_ = 36.7%). The item with the highest loading on the general factor stem from the altruism scale, namely, “I am empathic with those who are in need” (*λ* = 0.79). Generally, the mean standardized loading on the general factor for all items was relatively high (mean |*λ*| = 0.52, *SD* = 0.13; range = |0.24–0.79|) which means that on average measurement for the items of unconditional concern for others' welfare was coherent and consistent. Of note, almost all items of the compassion scale exhibited only very low (largely nonsignificant) loadings on the specific factor after the shared variance was partialled out.^4^

The general factor for conditional concern for others' welfare most strongly captured item variances of traits reflecting low conditional concern for others, that is, negative reciprocity norm endorsement (*ECV*_*G*_ = 89.6%) and vengefulness (*ECV*_*G*_ = 72.1%). That said, the hostility facet of the aggressiveness scale was less strongly absorbed by the general factor (*ECV*_*G*_ = 23.1%). In turn, the general factor accounted for relatively small amounts of variance in traits reflecting high conditional concern for others, that is, forgiveness (*ECV*_*G*_ = 43.1%) and positive reciprocity (*ECV*_*G*_ = 6.3%). The pattern of item loadings further indicated that the general factor reflected mostly (low) negative reciprocity: the item with the highest (negative) loading was “If someone treats you badly, you should treat that person badly in return” (*λ* = −0.87). Overall, the items displayed a moderately strong absolute loading on the general factor (mean |*λ*| = 0.45, *SD* = 0.25; range = |0.02–0.88|). Notably, the relatively high standard deviation of loadings indicates that the general factor captured the different traits to varying extents.

For the core tendency for self‐regulation, items from the impulsivity (*ECV*_*G*_ = 60.0%) and self‐control (*ECV*_*G*_ = 55.1%) scales shared the highest amounts of variance with the general factor. In turn, the general factor captured less shared variance with consideration for future consequences (*ECV*_*G*_ = 35.7%) and social responsibility (*ECV*_*G*_ = 36.0%). The items with the highest loadings were “I am able to work effectively toward long‐term goals.” (*λ* = 0.67) and “I concentrate easily.” (*λ* = 0.67) from the self‐control scale. The mean (absolute) standardized loading for the common core of self‐regulation was 0.39 (*SD* = 0.13; range = |0.13–0.67|) and, thus, descriptively smaller than for unconditional and conditional concern for others' welfare.

Lastly, the general factor for beliefs about others' pro‐sociality mostly captured item variances for general trust (*ECV*_*G*_ = 86.3%) and trust propensity (*ECV*_*G*_ = 57.6%). By contrast, the two traits capturing negative beliefs about others, that is, cynicism (*ECV*_*G*_ = 37.8%) and dangerous world view (*ECV*_*G*_ = 44.6%), shared smaller amounts of variance with the general factor. The lowest general factor saturation was apparent for reciprocity beliefs (*ECV*_*G*_ = 7.7%), which suggests that this trait was not well represented in the common core. The item “I am trustful.” (*λ* = 0.87) of the General Trust Scale yielded the highest loading. On average, items loaded moderately high on the general factor (mean |*λ*| = 0.42, *SD* = 0.26; range = |0.01–0.87|), while the relatively high standard deviation demonstrates that not all traits were captured equally well.

Overall, these results indicate that scales of the same trait class share a meaningful amount of variance, suggesting that there is an underlying common core (i.e., a core tendency). However, in all models, there was also meaningful variance over and above the general factor, reflecting item covariation that is not explained by the common core. On the one hand, this shows that there is a sufficient level of multidimensionality in the data to warrant a bifactor model instead of a unidimensional correlated factors model. On the other hand, while sharing meaningful variance, the specific items of each trait scale tap into unique aspects above and beyond the common core that are not shared with other related constructs.^5^

### 3.3 Latent regressions predicting pro‐social behavior

**FIGURE 1:**
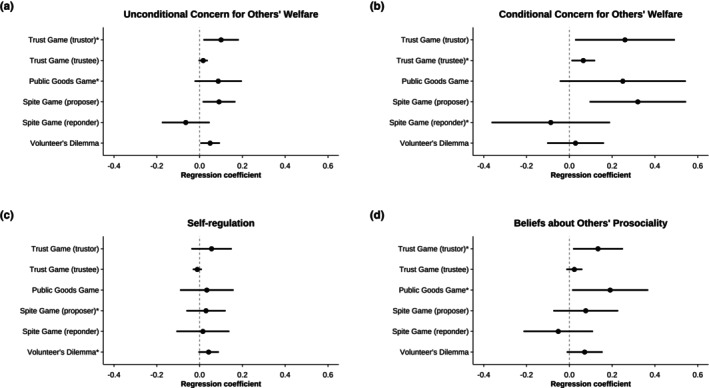
Regression coefficients for predicting pro‐social behavior outcomes by each core tendency. Pro‐social behavior in six economic games is predicted by the general factor (i.e., core tendency) of each bifactor model. Panel A: Results for unconditional concern for others’ welfare. Panel B: Results for conditional concern for others’ welfare. Panel C: Results for self‐regulation. Panel D: Results for beliefs about others’ prosociality. Error bars show 95% CIs. * indicate theorized relation between core tendency and behavioral outcome as per our hypotheses.

In our main analyses, we predicted pro‐social behavior in the different games by the respective core tendency as modeled in the baseline bifactor models. Results of these latent regression analyses are summarized in Table 5 with an overview for all core tendencies and all behavioral outcomes in the supplemental materials (Table S8). Figure 1 depicts the corresponding regression coefficients. Additionally, the statistical comparisons of the latent bivariate correlations according to our hypotheses are shown in Table 6.

**TABLE 5:** Results of latent regressions predicting pro‐social behavior outcomes by the respective core tendency.

Core tendency	*n*	*df*	Model fit		Outcome		
			RMSEA [90% CI]	SRMR	*b* [95% CI]	*R* ^2^	*p*
Unconditional concern for others' welfare	Trust Game (trustor)						
	361	1396 (156)	0.060 [0.057, 0.063]	0.069	0.100 [−0.018, 0.183]	0.017	0.017
	Public Goods Game						
	365	1396 (156)	0.056 [0.053, 0.059]	0.063	0.087 [−0.023, 0.197]	0.007	0.120
Conditional concern for others' welfare	Trust Game (trustee)						
	373	1223 (155)	0.053 [0.048, 0.056]	0.062	0.065 [0.010, 0.120]	0.004	0.020
	Spite Game (responder)						
	341	1223 (155)	0.052 [0.048, 0.056]	0.07	−0.087 [−0.363, 0.190]	0.008	0.538
Self‐regulation	Spite Game (proposer)						
	306	1079 (146)	0.067 [0.064, 0.071]	0.084	0.030 [−0.061, 0.121]	0.002	0.520
	Volunteer's Dilemma						
	314	1079 (146)	0.071 [0.068, 0.075]	0.086	0.042 [−0.005, 0.089]	0.011	0.079
Beliefs about others' pro‐sociality	Trust Game (trustor)						
	332	404 (92)	0.056 [0.049, 0.062]	0.068	0.131 [0.018, 0.250]	0.015	0.024
	Public Goods Game						
	321	404 (92)	0.052 [0.044, 0.058]	0.061	0.191 [0.013, 0.368]	0.018	0.035

Abbreviations: *b*, unstandardized regression coefficient; *df*, degrees of freedom (i.e., number of parameters); *n*, sample size; RMSEA, root mean square error of approximation; SRMR, standardized root mean square residual.

**TABLE 6:** Results for comparison of latent correlations as per our hypotheses.

Core tendency	Hypothesized relation with pro‐social behavior in …	*r*	*z*‐Test statistic for comparison of correlations					
			Trust Game (trustor)	Trust Game (trustee)	Public Goods Game	Spite Game (proposer)	Spite Game (responder)	Volunteer's Dilemma
Unconditional concern for others' welfare	Trust Game (trustor)	0.131		0.469		−0.090	0.807	0.267
	Public Goods Game	0.084		−0.169		−0.693	0.185	−0.359
Conditional concern for others' welfare	Trust Game (trustee)	0.065	−0.751		−0.516	−1.665		0.523
	Spite Game (responder)	−0.087	−0.441		−0.213	−1.350		0.805
Self‐regulation	Spite Game (proposer)	0.023	−0.637	1.033	−0.126		0.088	
	Volunteer's Dilemma	0.042	−0.398	1.284	0.114		0.328	
Beliefs about others' pro‐sociality	Trust Game (trustor)	0.134		0.727		0.792	1.240	0.332
	Public Goods Game	0.191		1.491		1.532	1.987*	1.081

*Note*: *r* = latent correlation between core tendency and economic games as per our hypotheses.

* *p* < 0.05 (one‐tailed).

As expected, unconditional concern for others' welfare was positively related to pro‐social behavior in the Trust Games as a trustor, yielding a small effect (*b* = 0.01, *p* = 0.017). However, it failed to significantly predict contributions in the Public Goods Game as hypothesized. The relation with trustees' pro‐social behavior did not result in a statistically significant association. To assess discriminant validity, we also computed the relations between unconditional concern for others' welfare and pro‐social behavior in the other games in which a possibility for exploitation is less salient or entirely absent. Contrary to expectations, there was a small positive relation between unconditional concern for others' welfare and proposer offers in the Spite Game (*b* = 0.09, *p* = 0.019). By contrast, there was no significant link between this core tendency and pro‐social behavior in the Spite Game as responder (*b* = −0.07, *p* = 0.256) and the Volunteer's Dilemma (*b* = 0.05, *p* = 0.058). However, the hypothesized effects did not significantly exceed the non‐hypothesized effects in size (see Table 6).

Regarding conditional concern for others' welfare, there was only weak support for the predictions we derived from the affordance‐based framework. Specifically, conditional concern for others' welfare weakly predicted trustees' behavior in the Trust Game (*b* = 0.07, *p* = 0.020) but failed to predict responders' behavior in the Spite Game (*b* = −0.09, *p* = 0.538). Unlike hypothesized, conditional concern for others was significantly associated with trustors' behavior in the Trust Game (*b* = 0.26, *p* = 0.029) and proposers' behavior in the Spite Game (*b* = 0.32, *p* = 0.005). Correspondingly, none of the *z*‐tests for the comparison of correlations supported our predictions (see Table 6).

Self‐regulation did not show a meaningful relation with pro‐social behavior in any of the games we hypothesized, that is, it neither accounted for variance in proposers' behavior in the Spite Game (*b* = 0.030, *p* = 0.520) nor in decisions in the Volunteer's Dilemma (*b* = 0.071, *p* = 0.079). In fact, self‐regulation showed no significant relation with pro‐social behavior in any of the games, that is, neither for trustors' transfers in the Trust Game (*b* = 0.056, *p* = 0.243), nor for trustees' returns in the Trust Game (*b* = −0.011, *p* = 0.311), nor for contributions in the Public Goods Game (*b* = 0.034, *p* = 0.597). By implication, there were also no statistically significant differences between the correlations in games involving temporal conflict and the games without this affordance.

Finally, more conclusive support in favor of our hypotheses was apparent for beliefs about others' pro‐sociality: This core tendency significantly predicted both the trustors' behavior in the Trust Game (*b* = 0.13, *p* = 0.024) and contributions in the Public Goods Game (*b* = 0.19, *p* = 0.035), as hypothesized. In turn, it did not significantly predict pro‐social behavior in any of the other games, that is, the Spite Game as proposer (*b* = 0.08, *p* = 0.320), the Spite Game as responder (*b* = −0.05, *p* = 0.531), and the Volunteer's Dilemma (*b* = 0.07, *p* = 0.094). However, most of the comparisons of hypothesized and non‐hypothesized correlations failed to reach a conventional level of statistical significance (see Tables 6 and S9).

Overall, there was generally mixed evidence for the propositions derived from the affordance‐based framework. Whereas the correlational pattern was in line with our predictions in many cases, the comparison of hypothesized versus non‐hypothesized correlations failed in almost all cases. As discussed in what follows, a plausible reason is a lack of statistical power for this set of statistical tests in particular.

## 4 DISCUSSION

Research showing consistent individual differences in the willingness to act pro‐socially—and corresponding links between personality traits and pro‐social behavior—is abundant. According to a recently proposed theoretical framework, the relations between different traits and pro‐social behavior in different social situations can be understood in terms of the features of those situations, so‐called affordances (Thielmann et al., 2020). Although this theoretical assumption has received some meta‐analytic support, a direct empirical test was as of yet missing. The goal of the present study was to close this gap and provide such a direct empirical test by studying the relation of a variety of personality traits with pro‐social behavior in six economic games involving the key situational affordances as per the theoretical framework under scrutiny. Specifically, we applied bifactor modeling to extract the shared variance of conceptually related traits to model four “core tendencies,” each of which was hypothesized to predict pro‐social behavior in the presence of a certain affordance. Overall, results were mixed, showing only some support for the affordance‐based framework.

### 4.1 Testing the theoretical account of individual differences in pro‐social behavior

The key premise of the affordance‐based framework (Thielmann et al., 2020) is that individual differences in behavior can be understood by the way personality traits are activated in response to four key situational affordances present in social situations. First, social situations are proposed to involve a possibility for exploitation that allows the expression of traits related to unconditional concern for others' welfare, such as altruism, empathy, and low exploitativeness. As hypothesized, the core tendency capturing unconditional concern for others' welfare was associated with higher transfers by trustors in the Trust Game and it also showed a positive link with proposers' generosity or fairness in the Spite Game which was beyond our predictions. Contrary to our assumption, the core tendency was not associated with Trustees' returned transfers in the Trust Game. Unlike predicted, however, unconditional concern for others was unrelated to contributions in the Public Goods Game, which also provides a possibility for exploitation. This finding contradicts meta‐analytic evidence showing small to medium‐sized relations between single traits capturing individual differences in unconditional concern for others (e.g., altruism, compassion, and honesty‐humility) and pro‐social behavior in social dilemma games, such as the Public Goods Game (Thielmann et al., 2020). Admittedly, we do not have a reasonable explanation for this unexpected finding. In any case, it cannot be attributed to the core tendency capturing something else than unconditional concern for others: altruism, empathy, and compassion—and, thus, traits that unequivocally capture unconditional concern for others' welfare—were those traits most strongly represented within this core tendency. Besides in the Trust Game as trustor, unconditional concern for others was also positively related to proposers' offers in the Spite Game. Given that responders can reject the proposer's offer in this game—in which case the proposer goes away empty‐handed—proposers should strategically give some of their endowment to the responder. Nonetheless, giving for strategic reasons may result in lower offers than giving based on high unconditional concern for others. Correspondingly, some traits related to unconditional concern for others (e.g., social value orientation, empathy, and honesty‐humility) also produced positive, albeit mostly small, meta‐analytic correlations with proposers' offers in the Ultimatum Game (Thielmann et al., 2020), which is highly similar to the Spite Game but in which responders also go away empty‐handed if they reject the proposer's offer. Thus, although not hypothesized, the finding can be reconciled with the affordance‐based framework. In turn, unconditional concern for others' welfare was unrelated to pro‐social behavior in all other games, as expected. Even though we presumed that the trustee in the Trust Game also faces a possibility for exploitation, we did not find a statistically significant link between the core tendency and trustees' pro‐social behavior. However, effect sizes did not differ significantly between games providing a possibility for exploitation and games involving other affordances. Overall, the results for unconditional concern for others' welfare in relation to pro‐social behavior were somewhat mixed, albeit being largely in line with the affordance‐based framework.

Second, social situations often provide a possibility for reciprocity, and in these situations, traits capturing conditional concern for others' welfare should be expressed. Confirming part of our hypotheses, we found a small positive relation between conditional concern for others' welfare and pro‐social behavior as measured by the trustees' return in the Trust Game. In contrast to our predictions, however, there was no evidence for conditional concern for others to significantly predict pro‐social behavior of responders in the Spite Game. Additionally, conditional concern for others was predictive in the Trust Game for trustors and the Spite Game for proposers, both of which do not provide a possibility for reciprocity. Thus, the hypothesized relations were also not significantly larger than the non‐hypothesized relations. Critically, an essential precondition of validly interpreting our findings in terms of the affordance‐based framework is that the core tendency we extracted indeed captures conditional concern for others' welfare. However, this was not readily the case: The general factor primarily represented negative reciprocity—the tendency to respond to others' actions with vengeful behaviors—rather than positive reciprocitythe tendency to return favors. In turn, the games we used to model a possibility for reciprocity were implemented using the strategy method; thus, participants did not directly react to others' behavior but simply anticipated how they would react to all conceivable actions by their interaction partner. In such situations, negative reciprocity is unlikely to unfold (Oosterbeek et al., 2004; Thielmann et al., 2021), thus arguably explaining the lack of evidence for our predictions.

Third, social situations are often characterized by a temporal conflict, meaning that short‐ and long‐term interests are at odds. In these situations, traits related to self‐regulation should manifest themselves in behavior. Contrary to this prediction, however, there was no indication for the core tendency of self‐regulation to predict pro‐social behavior in the presence of temporal conflict, which replicates the null effects found in prior meta‐analytic tests (Thielmann et al., 2020). That is, even in games specifically selected so as to elicit a conflict between short‐term and long‐term interest (e.g., the Volunteer's Dilemma), there was no relation between self‐regulation and pro‐social behavior. A plausible reason for the lack of effects in the current study is that the core tendency of self‐regulation did not readily capture individual differences in the ability to suppress one's impulses in the face of long‐term benefits. Instead, as evidenced by the items with the highest loadings (e.g., “I am able to work effectively toward long‐term goals”), the core tendency mostly represented individual differences in long‐term planning. Another explanation for the absence of evidence linking self‐regulation to pro‐social behavior is that self‐regulation may only be relevant for pro‐social behavior among dispositionally selfish individuals who have to suppress the immediate urge to behave in uncooperative ways (Moshagen et al., 2023; Yamagishi et al., 2017) suggesting an interaction with unconditional concern rather than a main effect of self‐regulation on pro‐social behavior. Finally, another possibility is that the affordance of temporal conflict might not be salient in the economic games we used. All that said, based on the current state of evidence, one may conclude that individual differences in self‐regulation are of little importance for the understanding of pro‐social behavior.

Finally, in many social situations, pro‐social behavior means to make oneself dependent on others' unknown behavior, suggesting that beliefs about others' pro‐sociality should guide behavior. In line with this prediction, positive beliefs about others were predictive of pro‐social behavior in both games characterized by dependence under uncertainty, that is, the Trust Game as trustor and the Public Goods Game. Effect sizes were rather small, though. No relation occurred with pro‐social behavior in any other game, as expected. Comparison of correlations further showed that the relation between beliefs about others' pro‐sociality and contributions in the Public Goods Game was significantly stronger than the correlation of this core tendency with responders' behavior in the Spite Game. All other relevant comparisons, however, turned out to be nonsignificant. Importantly, the extracted core tendency indeed represented beliefs about others' pro‐sociality well, showing the strongest link with trust propensity which capture confidence in others' trustworthiness.

Taken together, our findings show that personality traits do account for individual differences in pro‐social behavior in situations involving a possibility for exploitation and dependence on others under uncertainty. However, in situations characterized by temporal conflict, a systematic link with personality traits capturing individual differences in self‐regulation has yet to be shown. Finally, results for pro‐social behavior in situations providing a possibility for reciprocity remain inconclusive given the insufficient representation of positive reciprocity in the extracted core tendency intended to represent conditional concern for others' welfare.

### 4.2 Limitations and directions for future research

Although the approach taken in our study allowed us to extend previous work in various ways, some limitations ought to be acknowledged. First, all games were one‐shot, thus arguably increasing measurement error in our behavioral outcomes. Moreover, participants made their decisions simultaneously, even in games modeling a sequential decision process where one player reacts to another (e.g., Trust Game). To implement those games, choices of the reacting players (e.g., trustees) were measured using the strategy method, thus being somewhat hypothetical in nature. Moreover, most economic games—including the ones we relied on—do not isolate a specific affordance but, instead, involve multiple affordances, which may also vary in their saliency (Betsch et al., 2013). For example, although both the Spite Game and the Volunteer's Dilemma involve a tradeoff between immediate selfishness and potentially delayed gratification from pro‐sociality—and thus, temporal conflict—they also afford beliefs about others' pro‐sociality because one's own ultimate outcome also depends on the interaction partners' unknown behavior.

Second, we based our reasoning and hypotheses solely on theoretical considerations about which affordances should be present in a particular game (Thielmann et al., 2021). However, whether these are actually the affordances that individuals perceive is essentially unknown (Gerpott et al., 2021). More research on the perception of affordances is required to better understand the relation between personality traits and pro‐social behavior as a function of (perceived) situational characteristics.

Third, we only estimated the zero‐order relations with the core tendencies and pro‐social behavior in the different games while remaining mute on their incremental predictive validity over and above the respective other three core tendencies. However, due to the high model complexity and resulting convergence issues, it was not possible to estimate latent regression coefficients from multivariate models. We hope that this issue can be addressed in future research, for example, by means of novel analytic tools such as the structural after measurement approach (SAM) approach (Rosseel & Loh, 2022).

Finally, due to the between‐subjects assignment of games to participants, the sample sizes per game were relatively small (i.e., 391 ≤ *n* ≤ 452). For one, this is problematic due to the high complexity of the models that we estimated. As a consequence, some of the effects—though sizable in magnitude—failed to reach a conventional level of statistical significance, suggesting limited statistical power. Moreover, we had limited statistical power for the comparison of correlations. Assuming *n* = 400 per group, correlations would have needed to differ by at least Δ*r* = 0.17 to detect a significant difference with satisfactory power (1 − *β* = 0.80). However, correlations between personality traits and pro‐social behavior rarely exceed *r* = 0.20 and even the correlation between Social Value Orientation—the weights individuals assign to their own versus others' outcomes, which is usually measured in a series of the Dictator Games (Murphy et al., 2011)—and Dictator Game giving only amounts to *r* = 0.32 according to meta‐analytic estimates (Thielmann et al., 2020). Thus, finding differences in correlations was difficult to begin with, limiting the conclusiveness of the *z*‐tests. In fact, interaction effects—which we essentially tested here, though only indirectly via correlation comparisons—are often modest in size and thus require larger samples to obtain sufficient statistical power (Sommet et al., 2023). Future research using larger samples are thus needed to scrutinize the robustness of our results.

## 5 CONCLUSION

The present work once more highlights the importance of considering the overlap between situational affordances and personality traits and implies that the expression of traits is conditional on the affordances a situation provides. However, in the domain of pro‐social behavior as, for example, modeled in economic games, additional research is needed to further understand these person–situation transactions. This was particularly evident for the core tendency of self‐regulation, and less so for conditional concern for others' welfare. By and large, the current investigation extends prior findings on the link between personality and pro‐social behavior that has largely been based on zero‐order correlations by testing how the common core of various related traits manifests in pro‐social behavior. Nonetheless, further tests of the affordance‐based framework of individual differences in pro‐social behavior are required to potentially update the framework and get an even deeper understanding of the traits that drive pro‐social behavior in different social situations, including in daily life.

## AUTHOR CONTRIBUTIONS

Natalie Popov: Conceptualization, writing—original draft, formal analysis, visualization. Isabel Thielmann: Conceptualization, investigation, writing—reviewing and editing, supervision, project administration, funding acquisition.

## CONFLICT OF INTEREST STATEMENT

All authors declare that they have no conflicts of interest.

## ETHICS STATEMENT

The study was approved by the local ethics board at the University of Koblenz‐Landau (LEK‐308).

## Supporting information


Data S1.

## Data Availability

All materials, the data, analysis scripts, and supplementary results are available on the Open Science Framework (https://osf.io/cymxz/).

## ENDNOTES

^1^
In addition to the four key affordances, Thielmann et al. (2020) further proposed more specific subaffordances that may be present in situations providing possibilities for exploitation or reciprocity, respectively. Here, however, we focus on the four broad affordances and, therefore, refrain from further discussion of the subaffordances and the social motives they may activate.

^2^
In line with this reasoning, we excluded broad, basic personality traits (i.e., Big Five, HEXACO, Dark factor, and PID‐5 dimensions) because, by definition, broad traits subsume multiple aspects and are thus likely linked to multiple trait classes (Ones & Viswesvaran, 1996).

^3^
The trustee also faces a possibility for exploitation because the trustor cannot react to the trustee's decision. Although we did not preregister to use trustee behavior as an indicator of prosocial behavior in the presence of a possibility for exploitation (because we intended to have two hypotheses per core tendency), we investigated its link with unconditional concern for others' welfare in an exploratory fashion given that it also provides a valid test of the affordance‐based framework.

^4^
Arguably, this led to convergence issues in the bifactor model including the behavioral outcomes as criteria. Thus, we applied a different identification strategy in the latent regression models for unconditional concern for others' welfare by fixing latent variances to one.

^5^
To further test the legitimacy of the proposed underlying factor structure of four core tendencies, we followed the advice of an anonymous reviewer and additionally estimated a SEM including all core tendencies and their respective specific traits and assuming a simple structure. Model fit indices implied an excellent model fit (e.g., robust RMSEA = 0.035, 90% CI [0.034, 0.035], SRMR = 0.08), supporting that the data can indeed be well represented by four distinct, albeit interrelated, factors. Additional fit statistics as well as estimated factor intercorrelations are available in Tables S10 and S11 in the supplemental material.
