# Supplementary material for: The core tendencies underlying prosocial behavior: Testing a person–situation framework
Source: J Pers. 2024 Jul 2;93(3):633–52. doi: 10.1111/jopy.12957 (PMC12053821; doi:10.1111/jopy.12957)
Supplement: Supplementary file 1 — Data S1. [file JOPY-93-633-s001.docx]

**Supplemental Materials**

accompanying the manuscript “The Core Dispositional Tendencies Underlying Prosocial Behavior: Testing a Person-Situation Framework”

[**Descriptive Statistics** 2](#_Toc166495776)

[**Table S1.** *Descriptive Statistics of Personality Traits* 2](#_Toc166495777)

[**Table S2**. *Correlations with 95% Confidence Intervals for Traits Related to Unconditional Concern for Others’ Welfare* 3](#_Toc166495778)

[**Table S3***: Correlations with 95% Confidence Intervals for Traits Related to Conditional Concern for Others’ Welfare* 4](#_Toc166495779)

[**Table S4**. *Correlations with Confidence Intervals for Traits Related to Self-Regulation* 4](#_Toc166495780)

[**Table S5.** *Correlations with Confidence Intervals for Traits Related to Beliefs about Others’ Prosociality* 5](#_Toc166495781)

[**Bifactor Modelling** 6](#_Toc166495782)

[**Table S6.** *Bifactor Model Indices* 6](#_Toc166495783)

[**Table S7**. *Reference Indicator for the Baseline Bifactor Models and Decomposition of Explained Common Variance* 7](#_Toc166495784)

[**Table S8**. *Summary of Model Fit and Regressions for all Core Tendencies and all Economic Games* 9](#_Toc166495785)

[**Table S9.** *Comparison of Latent Correlations as per Our Hypotheses Using One-Sided z-Tests* 13](#_Toc166495786)

[**Table S10**. *Model Fit Indices for SEM Including all Core Tendencies* 14](#_Toc166495787)

[**Table S11**. *Estimated Latent Correlations between all Core Tendencies* 14](#_Toc166495788)

[**References** 14](#_Toc166495789)

# **Descriptive Statistics**

## **Table S1.** *Descriptive Statistics of Personality Traits*

| Core tendency | Narrow trait | *N* | *M* | *SD* |
| --- | --- | --- | --- | --- |
| Unconditional concern for others’ welfare | Altruism | 2,961 | 3.67 | .59 |
|  | Compassion | 3,045 | 3.24 | .76 |
|  | Empathy | 2,996 | 3.65 | .51 |
|  | Exploitation | 3,009 | 1.77 | .69 |
|  | Selfishness (facet: adaptive) | 3,047 | 2.74 | .71 |
|  | Selfishness (facet: egocentric) | 3,047 | 2.44 | .70 |
|  | Social welfare concerns | 3,053 | 3.46 | .69 |
| Conditional concern for others’ welfare | Aggressiveness (facet: hostility) | 2,980 | 2.46 | .70 |
|  | Forgiveness | 2,971 | 3.29 | .60 |
|  | Negative reciprocity norm endorsement | 2,978 | 2.46 | .72 |
|  | Positive reciprocity | 2,980 | 4.07 | .47 |
|  | Vengefulness | 2,952 | 2.15 | .76 |
| Self-regulation | Consideration of future consequences | 2,914 | 3.54 | .53 |
|  | Impulsivity | 2,823 | 2.43 | .51 |
|  | Self-control | 2,947 | 3.23 | .53 |
|  | Social responsibility | 2,951 | 4.21 | .47 |
| Beliefs about others’ prosociality | Cynicism | 2,704 | 3.39 | .73 |
|  | Dangerous world view | 2.642 | 2.89 | .78 |
|  | General trust | 2,563 | 3.47 | .66 |
|  | Reciprocity beliefs | 2,701 | 3.18 | .47 |
|  | Trust propensity | 2,697 | 3.40 | .76 |

*Note*. *N* = number of participants with complete data on respective trait within the Prosocial Personality Project; all personality traits were assessed on a 5-point Likert-scale from 1 = disagree to 5 = agree

**Unconditional Concern for Others’ Welfare**

## **Table S2**. *Correlations with 95% Confidence Intervals for Traits Related to Unconditional Concern for Others’ Welfare*

| Variable | 1 | 2 | 3 | 4 | 5 | 6 |
| --- | --- | --- | --- | --- | --- | --- |
| 1. Altruism |  |  |  |  |  |  |
|  |  |  |  |  |  |  |
| 2. Compassion | .72** |  |  |  |  |  |
|  | [.71, .74] |  |  |  |  |  |
|  |  |  |  |  |  |  |
| 3. Empathy | .74** | .67** |  |  |  |  |
|  | [.72, .75] | [.65, .69] |  |  |  |  |
|  |  |  |  |  |  |  |
| 4. Exploitativeness | -.47** | -.39** | -.54** |  |  |  |
|  | [-.50, -.44] | [-.42, -.36] | [-.56, -.51] |  |  |  |
|  |  |  |  |  |  |  |
| 5. Selfishness (adaptive) | -.43** | -.41** | -.45** | .58** |  |  |
|  | [-.46, -.40] | [-.44, -.38] | [-.48, -.42] | [.55, .60] |  |  |
|  |  |  |  |  |  |  |
| 6. Selfishness (egocentric) | -.67** | -.59** | -.62** | .62** | .69** |  |
|  | [-.69, -.65] | [-.61, -.56] | [-.64, -.59] | [.60, .64] | [.67, .71] |  |
|  |  |  |  |  |  |  |
| 7. Social Welfare Concerns | .50** | .46** | .47** | -.33** | -.31** | -.45** |
|  | [.48, .53] | [.43, .48] | [.44, .49] | [-.37, -.30] | [-.34, -.27] | [-.47, -.42] |
|  |  |  |  |  |  |  |

*Note.* 95% confidence intervals in brackets.

* *p* < .05, ** *p* < .01.

**Conditional Concern for Others’ Welfare**

## **Table S3***: Correlations with 95% Confidence Intervals for Traits Related to Conditional Concern for Others’ Welfare*

| Variable | 1 | 2 | 3 | 4 |
| --- | --- | --- | --- | --- |
| 1. Aggressiveness (hostility) |  |  |  |  |
|  |  |  |  |  |
| 2. Forgivingness | -.47** |  |  |  |
|  | [-.50, -.44] |  |  |  |
|  |  |  |  |  |
| 3. Negative reciprocity norm endorsement | .40** | -.59** |  |  |
|  | [.36, .43] | [-.61, -.56] |  |  |
|  |  |  |  |  |
| 4. Positive reciprocity | -.13** | .21** | -.18** |  |
|  | [-.17, -.10] | [.18, .25] | [-.21, -.14] |  |
|  |  |  |  |  |
| 5. Vengeance | .41** | -.58** | .76** | -.17** |
|  | [.38, .44] | [-.61, -.56] | [.75, .78] | [-.20, -.13] |
|  |  |  |  |  |

*Note.* 95% confidence intervals in brackets.

* *p* < .05, ** *p* < .01.

**Self-regulation**

## **Table S4**. *Correlations with Confidence Intervals for Traits Related to Self-regulation*

| Variable | 1 | 2 | 3 |
| --- | --- | --- | --- |
|  |  |  |  |
| 1. Consideration of future consequences |  |  |  |
|  |  |  |  |
| 2. Impulsivity | -.52** |  |  |
|  | [-.55, -.50] |  |  |
|  |  |  |  |
| 3. Self- control | .42** | -.63** |  |
|  | [.39, .45] | [-.66, -.61] |  |
|  |  |  |  |
| 4. Social responsibility | .36** | -.36** | .36** |
|  | [.33, .39] | [-.39, -.33] | [.32, .39] |
|  |  |  |  |

*Note.*95% confidence intervals in brackets.

* *p* < .05, ** *p* < .01.

.

**Beliefs about Others’ Prosociality**

## **Table S5.** *Correlations with Confidence Intervals for Traits Related to Beliefs about Others’ Prosociality*

| Variable | 1 | 2 | 3 | 4 |
| --- | --- | --- | --- | --- |
| 1. Cynicism |  |  |  |  |
|  |  |  |  |  |
| 2. Dangerous world view | .49** |  |  |  |
|  | [.46, .52] |  |  |  |
|  |  |  |  |  |
| 3. General trust | -.52** | -.56** |  |  |
|  | [-.54, -.49] | [-.59, -.53] |  |  |
|  |  |  |  |  |
| 4. Reciprocity beliefs | .12** | .09** | .05* |  |
|  | [.08, .15] | [.05, .13] | [.01, .08] |  |
|  |  |  |  |  |
| 5. Trust Propensity | -.30** | -.36** | .61** | .12** |
|  | [-.33, -.26] | [-.39, -.33] | [.59, .63] | [.08, .16] |
|  |  |  |  |  |

*Note.* 95% confidence intervals in brackets.

* *p* < .05, ** *p* < .01.

# **Bifactor Modeling**

## **Table S6.** *Bifactor Model Indices*

| Core tendency | PUC | ω | $\omega_{H}$ | H | FD |
| --- | --- | --- | --- | --- | --- |
| Unconditional concern for others’ welfare | .837 | .818 | .472 | .962 | .970 |
| Conditional concern for others’ welfare | .808 | .898 | .618 | .966 | .979 |
| Self-regulation | .754 | .771 | .228 | .915 | .942 |
| Beliefs about others’ prosociality | .811 | .712 | .062 | .935 | .951 |

*Note*. *PUC* = percent of uncontaminated correlations, indicates the number of unique correlations in a correlation matrix that are influenced by a single factor divided by the total number of unique correlations; ω = proportion of variance in the observed total score attributable to all “modelled” sources of common variance; $\omega_{H}$= percentage of variance in a unit-weighted composite that can be attributed to the general factor; *H* = construct replicability for all factors given standardized factor loadings (Hancock & Mueller, 2001); *FD* = factor determination, represents the correlation between factor score estimates and factors, all indices are calculated with the *BifactorIndices* package (Dueber, 2017)

## **Table S7**. *Reference Indicator for the Baseline Bifactor Models and Decomposition of Explained Common Variance*

| Core tendency | Reference indicator  (Scale) | Trait | $ECV_{GS}$ | $ECV_{SS}$ | $ECV_{SG}$ |
| --- | --- | --- | --- | --- | --- |
| Unconditional concern for others’ welfare | “I am pleased to help my friends/colleagues in their activities.”  (Altruism) | Altruism | .785 | .215 | .068 |
|  |  | Compassion | .792 | .208 | .024 |
|  |  | Empathy | .815 | .185 | .022 |
|  |  | Exploitativeness | .384 | .616 | .079 |
|  |  | Selfishness (adaptive) | .670 | .330 | .046 |
|  |  | Selfishness (egocentric) | .350 | .649 | .080 |
|  |  | Social welfare concerns | .368 | .632 | .040 |
| Conditional concern for others’ welfare | “I am ready to undergo personal costs to help somebody who helped me before.”  (Positive reciprocity) | Aggressiveness (facet: hostility) | .231 | .769 | .769 |
|  |  | Forgiveness | .431 | .569 | .087 |
|  |  | Negative reciprocity norm endorsement | .896 | .104 | .035 |
|  |  | Positive reciprocity | .063 | .937 | .144 |
|  |  | Vengefulness | .721 | .279 | .067 |

Table S7 *continued*

| Self-regulation | “I am good at resisting temptation.”  (Self-control) | Consideration of future consequences | .357 | .643 | .151 |
| --- | --- | --- | --- | --- | --- |
|  |  | Impulsivity | .600 | .401 | .129 |
|  |  | Self-control | .551 | .449 | .114 |
|  |  | Social responsibility | .360 | .640 | .121 |
| Beliefs about others’ prosociality | “Most people are basically honest.”  (Generalized trust) | Cynicism | .378 | .622 | .117 |
|  |  | Dangerous world view | .446 | .554 | .123 |
|  |  | General trust | .863 | .137 | .039 |
|  |  | Reciprocity beliefs | .077 | .923 | .128 |
|  |  | Trust propensity | .576 | .424 | .070 |

*Note*. $ECV_{GS}$ = proportion of common variance due to the general factor; $ECV_{SS}$ = proportion of common variance of the items of each specific factor due to the specific factor; $ECV_{SG}$ = proportion of common variance of all items due to the specific factor

.

## **Table S8**. *Summary of Model Fit and Regressions for all Core Tendencies and all Economic Games*

| Core tendency | *df* | Game | *n* | Model fit | | Outcome | | | | |
| --- | --- | --- | --- | --- | --- | --- | --- | --- | --- | --- |
|  |  |  |  | RMSEA [90% CI] | SRMR | *b*  [95% CI] | β | *SE* | *R*² | *p* |
| Unconditional concern for others’ welfare | 1,329 (156) | PGG | 365 | .056  [.053, .059] | .063 | .087  [-.023, .197] | .084 | .056 | .007 | .120 |
|  |  | TG1 | 362 | .060  [.057, .063] | .069 | .100  [.018, .183] | .131 | .042 | .017 | .017 |
|  |  | TG2 | 344 | .060  [.056, .063] | .081 | .017  [-.012, .113] | .096 | .011 | .010 | .115 |
|  |  | SG1 | 292 | .063  [.059, .066] | .076 | .091  [.015, .167] | .138 | .039 | .019 | .019 |
|  |  | SG2 | 331 | .062  [.059, .066] | .078 | -.065  [-.176, .047] | -.070 | .057 | .005 | .256 |
|  |  | VD | 336 | .057  [.053, .051] | .072 | .045  [-.001, .092] | .111 | .024 | .012 | .058 |

Table S8 *continued*

| Core tendency | df | Game | n | Model fit | | Outcome | | | | |
| --- | --- | --- | --- | --- | --- | --- | --- | --- | --- | --- |
|  |  |  |  | RMSEA [90% CI] | SRMR | *b*  [95% CI] | *β* | *se* | *R²* | *p* |
| Conditional concern for others’ welfare | 1,223 (155) | PGG | 360 | .052  [.048, .054] | .061 | .250  [-.044, .544] | .103 | .150 | .011 | .096 |
|  |  | TG1 | 366 | .053  [.049, .056] | .062 | .260  [.027, .493] | .120 | .119 | .014 | .029 |
|  |  | TG2* | 373 | .053  [.048, .056] | .062 | .065  [.010, .120] | .139 | .004 | .019 | .020 |
|  |  | SG1 | 322 | .052  [.048, .055] | .067 | .320  [.095, .128] | .190 | .115 | .036 | .005 |
|  |  | SG2* | 341 | .052  [.048, .056] | .070 | -.087  [-.363, .190] | -.034 | .141 | .001 | .538 |
|  |  | VD | 334 | .052  [.048, .056] | .069 | .029  [-.103, .161] | .025 | .068 | .001 | .668 |

Table S8 *continued*

| Core tendency | df | Game | n | Model fit | | Outcome | | | | |
| --- | --- | --- | --- | --- | --- | --- | --- | --- | --- | --- |
|  |  |  |  | RMSEA [90% CI] | SRMR | *b*  [95% CI] | *β* | *se* | *R²* | *p* |
| Self-regulation | 1,079 (146) | PGG | 330 | .071 [.067,.074] | .  .080 | .034  [-.091, .159] | .033 | .064 | <.001 | .597 |
|  |  | TG1 | 349 | .074  [.072, .076] | .081 | .056  [-.038, .150] | .073 | .048 | .005 | .243 |
|  |  | TG2 | 353 | .072  [.069, .075] | .089 | -.011  [-.031, .010] | -.058 | .011 | .003 | .311 |
|  |  | SG1* | 306 | .067  [.064, .071] | .084 | .030  [-.061,.121] | .044 | .047 | .002 | .520 |
|  |  | SG2 | 328 | .063  [.06, .067] | .074 | .015  [-.108, .139] | .016 | .063 | <.001 | .806 |
|  |  | VD* | 314 | .071  [.068, .075] | .086 | .042  [-.005, .089] | .106 | .024 | .011 | .079 |

Table S8 *continued*

| Core tendency | *df* | Game | *n* | Model fit | | Outcome | | | | |
| --- | --- | --- | --- | --- | --- | --- | --- | --- | --- | --- |
|  |  |  |  | RMSEA  [90% CI] | SRMR | *b*  [95% CI] | *β* | *se* | R² | *p* |
| Beliefs about others’ prosociality | 404 (92) | PGG* | 321 | .052  [.045, .058] | .061 | .191  [.013,.368] | .133 | .091 | .018 | .035 |
|  |  | TG1* | 332 | .056  [.049, .062] | .068 | .134 [.018,.250] | .123 | .059 | .015 | .024 |
|  |  | TG2 | 345 | .053  [.047, .059] | .060 | .023  [-.014, .060] | .077 | .019 | .006 | .215 |
|  |  | SG1 | 304 | .058  [.051, .064] | .070 | .077  [-.074, .228] | .070 | .077 | .005 | .320 |
|  |  | SG2 | 315 | .050  [.043, .057] | .053 | -.052  [-.214, .110] | -.035 | .083 | .001 | .531 |
|  |  | VD | 317 | .058  [.052, .064] | .069 | .071  [-.012, .155] | .107 | .043 | .011 | .094 |

*Note*. *df* = degrees of freedom (number of parameters), *n* = sample size, RMSEA = root mean square error of approximation, SRMR = standardized root mean square residual, *b* = unstandardized regression coefficient, β*=* standardized regression coefficient, *SE* = standard error, *R*² = coefficient of determination, PGG = Public Goods Game, TG1 = Trust Game (trustor), TG2 = Trust Game (trustee), SG1 = Spite Game (proposer), SG2 = Spite Game (responder), VD = Volunteer’s Dilemma, * relation as per our hypotheses.

## **Table S9.** *Comparison of Latent Correlations as per Our Hypotheses Using One-Sided z-Tests*

| Core tendency | Hypothesized relation | | | Public Goods Game | | Trust Game (trustor) | | Trust Game (trustee) | | Spite Game (proposer) | | Spite Game (responder) | | Volunteer’s Dilemma | |
| --- | --- | --- | --- | --- | --- | --- | --- | --- | --- | --- | --- | --- | --- | --- | --- |
|  | Game | | *r* | *z* | *p* | *z* | *p* | *z* | *p* | *z* | *p* | *z* | *p* | *z* | *p* |
| Unconditional concern for others’ welfare | PGG | | .084 | - | - | - | - | -.169 | .563 | -.693 | .756 | .185 | .427 | -.359 | .640 |
|  | TG1 | | .131 | - | - | - | - | .469 | .320 | -.090 | .536 | .807 | .210 | .267 | .395 |
| Conditional concern for others’ welfare | TG2 | .065 | | -.516 | .697 | -.751 | .774 | - | - | -1.665 | .952 | - | - | .523 | .298 |
|  | SG2 | -.087 | | -.213 | .584 | -.441 | .671 | - | - | -1.350 | .911 | - | - | .805 | .211 |
| Self-regulation | SG1 | | .023 | -.126 | .545 | -.637 | .738 | 1.033 | .151 | - | - | .088 | .465 | - | - |
|  | VD | | .042 | .114 | .455 | -.398 | .655 | 1.284 | .100 | - | - | .328 | .371 | - | - |
| Beliefs about others’ prosociality | PGG | | .191 | - | - | - | - | 1.491 | .068 | .1532 | .063 | 1.987 | .023 | 1.081 | .140 |
|  |  | |  |  |  |  |  |  |  |  |  |  |  |  |  |
|  | TG1 | | .134 | - | - | - | - | .727 | .234 | .792 | .214 | 1.240 | .108 | .332 | .370 |

*Note*. *r* = correlation coefficient, *z* = z-test statistic, PGG = Public Goods Game, TG1 = Trust Game (trustor), TG2 = Trust Game (trustee), SG1 = Spite Game (proposer), SG2 = Spite Game (responder), VD = Volunteer’s Dilemma

## **Table S10**. *Model Fit Indices for SEM Including all Core Tendencies*

| $\boldsymbol{\chi}^{\boldsymbol{2}}$  **(*df*)** | **RMSEA**  **[90% CI]** | **SRMR** | **CFI** |
| --- | --- | --- | --- |
| 46,313.31 (16,106) | .035  [.034, .036] | .080 | .71 |

*Note.* We assumed simple structure for all factor loadings (i.e., no cross-loadings) and set all specific factors to orthogonality

## **Table S11**. *Estimated Latent Correlations between all Core Tendencies*

|  | **Unconditional concern for others’ welfare** | **Conditional concern for others’ welfare** | **Self-regulation** |
| --- | --- | --- | --- |
| Unconditional concern for others’ welfare |  |  |  |
| Conditional concern for others’ welfare | .517** |  |  |
| Self-regulation | .194** | .230** |  |
| Belief about others’ prosociality | .383** | .478** | .193** |

*Note.* ** *p* < .01. All latent variances were set to 1

**References**

Dueber, D. M. (2017). *Bifactor Indices Calculator* [Computer software]. University of Kentucky Libraries.

Hancock, G. R., & Mueller, R. O. (2001). Rethinking Construct Reliability Within Latent Variable Systems. *Structural Equation Modeling: Present and Future*, *195*.
